# Soybean *GmbZIP123* gene enhances lipid content in the seeds of transgenic *Arabidopsis* plants

**DOI:** 10.1093/jxb/ert238

**Published:** 2013-08-20

**Authors:** Qing-Xin Song, Qing-Tian Li, Yun-Feng Liu, Feng-Xia Zhang, Biao Ma, Wan-Ke Zhang, Wei-Qun Man, Wei-Guang Du, Guo-Dong Wang, Shou-Yi Chen, Jin-Song Zhang

**Affiliations:** ^1^State Key Lab of Plant Genomics, Institute of Genetics and Developmental Biology, Chinese Academy of Sciences, Beijing 100101, PR China; ^2^University of Chinese Academy of Sciences, Beijing 100049, PR China; ^3^Institute of Soybean Research, Heilongjiang Provincial Academy of Agricultural Sciences, Harbin 150086, PR China

**Keywords:** Cell-wall intertase, *GmbZIP123* overexpression, seed lipid, soybean, sugar transport, sucrose transporter.

## Abstract

Soybean is one of most important oil crops and a significant increase in lipid content in soybean seeds would facilitate vegetable oil production in the world. Although the pathways for lipid biosynthesis in higher plants have been uncovered, our understanding of regulatory mechanism controlling lipid accumulation is still limited. In this study, we identified 87 transcription factor genes with a higher abundance at the stage of lipid accumulation in soybean seeds. One of these genes, *GmbZIP123*, was selected to further study its function in regulation of lipid accumulation. Overexpression of *GmbZIP123* enhanced lipid content in the seeds of transgenic *Arabidopsis thaliana* plants. The *GmbZIP123* transgene promoted expression of two sucrose transporter genes (*SUC1* and *SUC5*) and three cell-wall invertase genes (*cwINV1*, *cwINV3*, and *cwINV6*) by binding directly to the promoters of these genes. Consistently, the cell-wall invertase activity and sugar translocation were all enhanced in siliques of *GmbZIP123* transgenic plants. Higher levels of glucose, fructose, and sucrose were also found in seeds of *GmbZIP123* transgenic plants. These results suggest that *GmbZIP123* may participate in regulation of lipid accumulation in soybean seeds by controlling sugar transport into seeds from photoautotrophic tissues. This study provides novel insights into the regulatory mechanism for lipid accumulation in seeds and may facilitate improvements in oil production in soybean and other oil crops through genetic manipulation of the *GmbZIP123* gene.

## Introduction

As an important source of edible oils and industrial materials, vegetable oils exert broad functions in many aspects of human life. Due to enhanced demand of food use and biodiesel, the consumption and price of vegetable oils have both sharply increased in the past few years (Lu *et al*., 2011). Soybean oil is one of the most widely consumed edible oils, and an increase in oil content in soybean seeds would dramatically contribute towards reducing the shortage of vegetable oil supply worldwide. Given that the highest seed oil content is about 23% in soybean germplasm resources, it is a challenge to drastically increase the oil content of soybean seeds using traditional breeding and genetic engineering methods (Gai, 2003).

Vegetable oils, mainly in the form of triacylglycerols (TAGs), are synthesized during seed development in oilseed plants (Baud *et al*., 2008). They are the major energy storage in the seeds and are used to support successful seedling establishment after germination. Rapid lipid biosynthesis in seeds requires adequate carbohydrates, which are imported into seeds from photoautotrophic tissues, such as leaves and silique walls. As the major carbon source, sucrose is imported into the cells of seeds through two pathways. Sucrose can be cleaved to hexoses by cell-wall invertases (cwINVs) and the hexoses are then transported into cells through hexose transporters. Alternatively, sucrose is directly imported into cells through sucrose transporters (SUCs) (Shiratake, 2007). Disruption of sucrose import would weaken the lipid synthesis ability of seeds, or even influence seed development. In the *Arabidopsis* (*Arabidopsis thaliana*) mutant of *AtSUC5*, a SUC gene, fatty acid (FA) concentration is reduced strongly in seeds at 8 d after flowering and is 2–13% lower in dry seeds than that in wild-type seeds (Baud *et al*., 2005). Furthermore, a slight development delay in the embryo is also observed in *suc5* mutants. A recent study discovered that SUC5 can transport biotin and affect triacylglycerol accumulation (Pommerrenig *et al*., 2013). Loss-of-function mutations in the maize *cwINV2* gene lead to a drastic reduction in endosperm weight and size relative to that of the wild-type plants (Miller and Chourey, 1992; Cheng *et al*., 1996). Imported sucrose is cleaved to UDP-glucose and fructose by sucrose synthase in the cytoplasm. Hexose is further converted into pyruvate by glycolytic activity within the cytoplasm and the plastid. It has been found that intermediates of sugar metabolism such as glucose-6-phosphate and pyruvate are then imported into plastid from the cytoplasm (da Silva *et al*., 1997; Furumoto *et al*., 2011). Pyruvate dehydrogenase complex in plastids can transform pyruvate into acetyl-CoA, which is the substrate for *de novo* synthesis of FAs. Besides acetyl-CoA, sugar metabolism such as the pentose phosphate pathway also provides reducing power for FA biosynthesis, thus indicating that sugar metabolism is highly bound to FA biosynthesis.

In higher plants, *de novo* synthesis of FAs from acetyl-CoA in plastids is achieved by a series of catalysing reactions, including carboxylation of acetyl-CoA to malonyl-CoA by acetyl-CoA carboxylase (ACCase) and consecutively adding two carbons of malonyl-CoA to acyl chains by a FA synthase (FAS) complex (Ohlrogge and Browse, 1995; Rawsthorne, 2002). ACCase is a rate-limiting enzyme for FA synthesis, resulting in much lower concentrations of malonyl-CoA in contrast to acetyl-CoA, which limits the synthesis speed of FAs (Sasaki and Nagano, 2004). C16:1 and C18:1 mono-unsaturated FAs are produced through desaturation of FAs, which are catalysed by plastidial stearoyl-acyl carrier protein (ACP) desaturase. After elongation termination of plastidial FAs of up to 18 carbons in length, free FAs are exported to the endoplasmic reticulum and can be further extended to long-chain FAs by FA elongase (Ohlrogge and Browse, 1995; Voelker and Kinney, 2001). FAs are eventually esterified to glycerol 3-phosphate to form TAGs through the Kennedy pathway, which are catalysed by glycerol 3-phosphate acyltransferase, lysophosphatidic acid acyltransferase, and diacylglycerol acyltransferase (Kennedy, 1961; Settlage *et al*., 1998).

As the enzymes participating in FA biosynthesis have been thorough studied, many efforts to increase lipid production of seeds have been made through elevated expression of key enzymes. However, overexpression of a single gene in the ACCase or FAS complex could not significantly increase the flux through FA biosynthesis (Shintani *et al*., 1997; Dehesh *et al*., 2001). It seems likely that an increase in lipid accumulation in seeds requires upregulation of multiple related genes involved in carbon metabolism, FA synthesis, and end- product synthesis of lipid from FAs. In the past few years, several important transcription factors (TFs) have been found to participate in the regulation of seed maturation and lipid accumulation, including the basic leucine zipper (bZIP), B3, NF-YB, and DOF TF families (Focks and Benning, 1998; Tsukagoshi *et al*., 2007; Wang *et al*., 2007; Mu *et al*., 2008). Ectopic expression of *AtLEC1*, encoding an NF-YB TF, enhances expression of several lipid synthesis-related genes and increases the lipid content of leaves in *Arabidopsis* (Meinke *et al*., 1994; Mu *et al*., 2008). Mutants of *AtWRI1* show an 80% reduction in seed TAGs compared with wild-type plants and AtWRI1 is an AP2 TF (Focks and Benning, 1998; Baud *et al*., 2007). Besides positive regulators of lipid accumulation, negative regulators are also involved in lipid synthesis, such as *AtVAL1* and *AtVAL2* (Suzuki *et al*., 2007; Tsukagoshi *et al*., 2007). *AtVAL1* and *AtVAL2* belong to the B3 family of TFs. In *Arabidopsis val1 val2* double mutants, seedlings exhibit embryo-associated traits including accumulation of seed storage proteins and TAGs.

Previously, we found that soybean GmDOF4 and GmDOF11 promote lipid accumulation in seeds of transgenic plants through activation of FA biosynthesis genes but reduction of storage protein genes (Wang *et al*., 2007). In this study, we adopted RNA-seq technology to identify genes with high abundance in developing soybean seeds at the stage of rapid lipid accumulation. In total, 87 TFs were selected and the functions of these TFs in lipid accumulation were studied in transgenic *Arabidopsis* plants. We found that elevated expression of a TF gene, *GmbZIP123*, could enhance lipid content in the seeds of transgenic *Arabidopsis* plants. Expression of sucrose transporter genes (*SUC1* and *SUC5*) and cell-wall invertase genes (*cwINV1*, *cwINV3*, and *cwINV6*) was upregulated in *GmbZIP123* transgenic plants. Cell-wall invertase activity and sugar transportation also increased. More imported sugar in seeds may be responsible for the lipid content increase. Our study provides a novel insight into the regulation of lipid accumulation in soybean seeds, and genetic manipulation of the gene may have potential benefits in improvement of soybean oil production.

## Materials and methods

### Plant materials and growth conditions

Soybean (*Glycine max*) seeds of cultivar Heinong44 were planted directly in the experimental station in Beijing from May to August. Roots, stems, leaves, silique walls, and seeds at the indicated stages were harvested and stored at –70 °C for RNA isolation.

*Arabidopsis* plants were grown in a growth chamber at 22 °C with a photoperiod of 16h/8h (light/dark) per day. Seedlings were harvested from *Arabidopsis* plants at 3 weeks after germination and stored at –70 °C for RNA isolation.

### Quantitative reverse transcriptase-PCR (qRT-PCR)

Total RNA was isolated from plant tissues using TRIzol reagent (Invitrogen) according to the manufacturer’s instructions. First-strand cDNA was produced from 1 µg of total RNA using oligo(dT) as a primer and SuperScript II reverse transcriptase (Invitrogen). The cDNA was used as template for qRT-PCR using SYBR qPCR mix (Toyobo). The reaction was run on a LightCycler 480 System (Roche). The relative expression level was quantified using an internal control. The *TUBULIN* (GenBank accession no. XM_003520891) gene and *AtACTIN2* gene were selected as internal controls for soybean and *Arabidopsis* genes, respectively. All primers for qRT-PCR are listed in Supplementary Table S3 at *JXB* online.

### Plasmid construction and plant transformation

The coding region of *GmbZIP123* (*Glyma06g01240*) was PCR amplified using KOD DNA polymerase (KOD FX; Toyobo) and the primers (5′-GGATCCATGACTATGGCTTGTTCAAG-3′ and 5′-GGT ACCTCAGTACTGCAACATGTCTG-3′), subsequently cloned into the cauliflower mosaic virus 35S promoter-driven plant binary vector pROK2 using *Bam*HI/*Kpn*I sites. The constructed plasmid was confirmed by sequencing and transformed into *Agrobacterium tumefaciens* strain GV3101. The transformation of *Arabidopsis* plants was performed using the floral dip method.

### RNA-linker-mediated rapid amplification of 5′ cDNA ends (5′RACE)

Total RNA (200 µg) from soybean seeds was used to purify mRNA using an Oligotex kit (Qiagen). The poly(A) RNA was dephos phorylated by calf intestinal alkaline phosphatase (New England Biolabs), followed by tobacco acid pyrophosphatase treatment for decapping the RNA (Epicentre). The 5′ RNA adaptor (5′-CGACUGGAGCACGAGGACACUGACAUGGACUGAA GGAGUAGAAA-3′) was ligated to the decapped RNA by T4 RNA ligase (Ambion), followed by a reverse transcription reaction. The reverse transcription product was amplified using 5′ RNA adaptor primer (5′-GCACGAGGACACTGACATGGACTGA-3′) and a gene-specific primer (5′-TCAGCTGCGATGCTAGATC ATC-3′) for 30 cycles of PCR. Twenty-five cycles of PCR were further performed with the above PCR product as template, using a nested gene-specific primer (5′-TGATCCATCAGACCCTGCAGCT-3′) and a nested adapter primer (5′-GGACACTGACATGGACTG AAGGAGTA-3′). The final PCR product was detected by gel electrophoresis and extracted for sequencing.

### Phylogenetic analysis

The protein sequences of GmbZIP123 and *Arabidopsis* group S bZIP TFs were aligned using ClustalX software. The evolutionary analyses were conducted with 1000 bootstrap replicates in MEGA5, using the neighbour-joining method (Tamura *et al*., 2011).

### Subcellular localization of *GmbZIP123* in *Arabidopsis* protoplast

The coding region of *GmbZIP123* was cloned into the green fluorescent protein (GFP) vector GFP221 to generate the 35S:GmbZIP123–GFP construct using specific primers containing *Bam*HI and *Sal*I sites. The GFP221 vector containing the 35S:GFP construct was used as control. These plasmids were introduced into *Arabidopsis* protoplasts by polyethylene glycol-mediated transfection. Transfected cells were cultivated for 20h and then observed under a Leica TCS SP5 microscope.

### Transcriptional activation test in *Arabidopsis* protoplasts

Analysis of the transcriptional activation activity of GmbZIP123 in *Arabidopsis* protoplasts was performed as described previously (Wei *et al*., 2009). In brief, the coding region of *GmbZIP123* was cloned into the pRT-BD vector to generate 35S:GAL4BD–GmbZIP123 construct. The 35S:GAL4BD construct and 35S:GAL4BD–VP16 construct were selected as negative and positive controls, respectively. A plasmid containing a 5×GAL4–luciferase (LUC) construct was used as a reporter plasmid. These constructs were introduced into *Arabidopsis* protoplasts by polyethylene glycol-mediated transfection. After culturing for 16h, luciferase activity was assayed for each co-transfection sample using a GloMax^TM^ 20/20 Luminometer (Promega).

### Yeast two-hybrid analysis

Yeast two-hybrid analysis was performed as described previously (Wei *et al*., 2009). DNA binding (BD) and activation domain (AD) constructs were generated by inserting the coding regions of *Arabidopsis* group C bZIP TFs and GmbZIP123 into pAD-GAL4-2.1 and pBD-GAL4 vectors (Stratagene) as follows: pAD-AtbZIP9, pAD-AtbZIP10, pAD-AtbZIP25, pAD-AtbZIP63, and pBD-GmbZIP123. To analyse the transcriptional activation activity of GmbZIP123 by interacting with AtbZIP10 and AtbZIP25 in yeast, the coding regions of AtbZIP10 and AtbZIP25 were inserted into the pAUR123 vector (Takara). The pairs of relevant constructs were co-transformed into the yeast strain YRG2 to examine expression of a histidine (*HIS3*) reporter gene.

### RNA-seq library construction and sequencing data processing

An RNA-seq library was constructed following the Illumina kit recommendation. Briefly, poly(A) containing RNA was extracted from 15 µg of total RNA using an Oligotex kit. Poly(A)-containing RNA was fragmented and used as template for first-strand cDNA synthesis using reverse transcriptase and random primers, followed by second-strand cDNA synthesis. End repair and adding an A base to the 3′ end were performed on the double-stranded cDNA, and DNA adapter was then ligated to the DNA fragment. Finally, the adapter-ligated DNA was enriched by 15 cycles of PCR and gel purified for Illumina single-end sequencing.

After adapter clipping and quality filter, reads were mapped to the soybean genome sequence (Williams 82) using BWA software with no more than two nucleotide mismatches (Li and Durbin, 2009). Only reads mapping to unique locations were reserved. The expression abundance of genes was calculated using only uniquely mapped reads. Gene expression was normalized to reads per kb per million reads.

### Quantitative analysis of FA content

For methylation, 10mg of seeds and 100 µg of internal standard (heptadecanoic acid) were added to extraction solution (2.5%, v/v, H_2_SO_4_ in CH_3_OH) and incubated at 85 °C for 1h. After centrifugation, 500 µl of the supernatant was mixed with 600 µl of 0.9% (w/v) NaCl and 300 µl of hexane. The mixture was centrifuged at 4000rpm for 10min and the organic phase was dried by natural volatilization. FA methyl esters were dissolved with 50 µl of ethyl acetate and subjected to gas chromatography (GC) analysis (GC-2014; Shimadzu).

### Determination of sugar content

Ten milligrams of fine powder prepared from silique walls or 50 seeds was extracted in 80% (v/v) ethanol twice at 80 °C for 45min. The combined supernatants were evaporated to dryness in a stream of nitrogen gas. The derivatization of dry residues was performed using 100 µl of trimethylsilylimidazole and 100 µl of pyridine at 70 °C for 30min. Finally, 1 µl of derivatives was subjected to GC analysis as above.

### Measurement of cell-wall invertase activities

Samples of leaf and silique, which consisted of 200mg from 3- and 5-week-old Columbia 0 (Col-0) and *GmbZIP123* transgenic plants, respectively, were freshly collected and frozen in liquid nitrogen. CwINV activity was measured as described by Tomlinson *et al*. (2004). Experiments were done with four biological replicates.

### Labelling with ^13^CO_2_ and ^13^C enrichment analysis

Labelling was done in a growth chamber at 22 °C with a photoperiod of 16h/8h (light/dark) per day. Five-week-old Col-0 and *GmbZIP123* transgenic plants were placed in a plastic box (30×50cm) sealed with transparent lid. ^13^CO_2_ was generated by injecting 10ml of 1M HClO_4_ (Sigma) into a beaker with 0.5g of barium carbonate-^13^C (98 atom % ^13^C; Sigma). The plants were labelled for 1 d. Plants were then taken out and grown for an additional 1 d. Siliques at 5–15 d after flowering were collected for extraction and measurement of ^13^C-labelled sugar. All ^13^C measurements were performed using an Agilent 7890A-5975C GC-mass spectrometry system (Agilent Technologies). For GC/electron impact mass spectrometry, the levels of all metabolites and the ion abundance for each metabolite were quantified as described by Tan *et al*. (2011). ^13^C enrichment was calculated by the following equation:


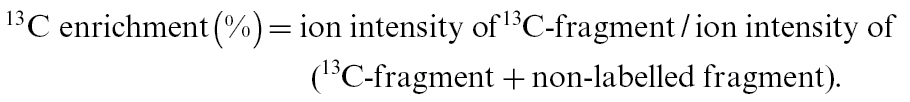


The characterized ions (labelled and non-labelled) for fructose, glucose, and sucrose were selected based on work published previously (Huege *et al*., 2007).

### Transactivation of target promoter by *GmbZIP123* in tobacco leaves

The 1.2kb sequences upstream from the ATG codons of *SUC1*, *SUC5*, *cwINV1*, *cwINV3*, and *cwINV6* were inserted into pGWB435 to generate promoter:LUC reporter constructs using Gateway^®^ technology (Invitrogen). The reporter plasmid and the pROK2 plasmid containing the 35S:GmbZIP123 construct were transformed into *Agrobacterium tumefaciens* strain GV3101. The *Agrobacterium* strains were incubated in Luria–Bertani medium and finally resuspended in infiltration buffer (10mM MES, 0.2mM acetosyringone, 10mM MgCl_2_) to an ultimate concentration of OD_600_=1.0. Equal amounts of different combined bacterial suspensions were infiltrated into the young leaves of the 5-week-old tobacco plants using a needleless syringe. After infiltration, the plants were grown first in the dark for 12h and then with 16h light/8h dark for 48h at 24 °C before charge-coupled device imaging. The leaves were sprayed with 100 µM luciferin (Promega) and placed in the dark for 5min. LUC activity was observed with a low-light cooled charge-coupled device imaging apparatus (iXon; Andor Technology). Experiments were performed with three independent biological replicates.

### Gel-shift assay

The recombinant protein of glutathione *S*-transferase–GmbZIP123 was expressed in *Escherichia coli* BL21(DE3) using avpGEX-6p-1 vector and purified from cells using Sepharose^TM^ Fast Flow (GE Healthcare). The examined fragments in promoters of target genes were produced from hybridization of synthesized oligonucleotides. The sequences of oligonucleotides are listed in Supplementary Table S4 at *JXB* online. The gel-shift assay was performed using a DIG Gel Shift kit (Roche) according to the manufacturer’s instructions.

## Results

### Identification of candidate genes relevant to lipid synthesis

**Fig. 1.**
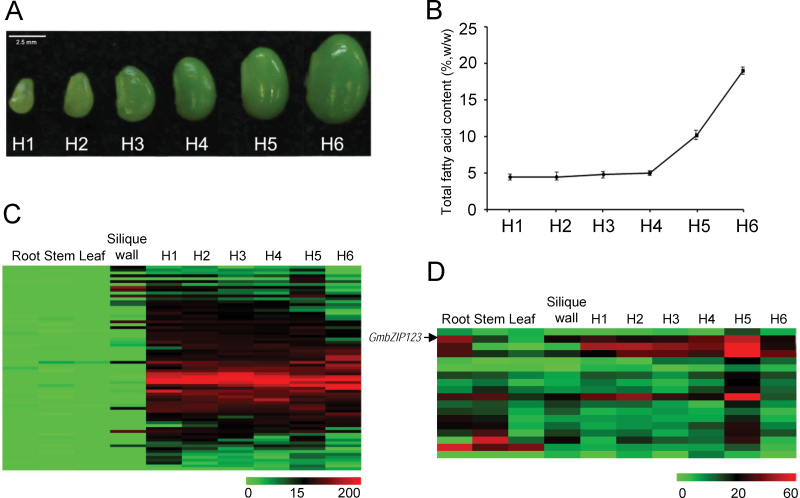
Identification of candidate TFs related to lipid accumulation. (A) Six developmental stages of soybean seeds. (B) Lipid content of seeds at different developmental stages. For each point, results indicate means±standard deviation (SD) (*n*=4). (C) Expression pattern of seed-preferred TFs in different soybean tissues. (D) Expression pattern of TFs preferentially expressed at stage H4 or H5 compared with those at other seed developmental stages.

Six developmental stages of soybean seeds were selected according to the ratio of seed weight of the stage to highest seed fresh weight, namely 4, 8, 12, 20, 35, and 80% (Fig. 1A; H1–H6). We examined the lipid content of seeds at all stages and found that FA accumulation increased dramatically at stage H5 (Fig. 1B). Lipid content changes in seeds were almost indistinguishable before stage H4.

RNA-seq was adopted to determine the gene-expression pattern in seeds at all six developmental stages and other organs including roots, leaves, stems, and silique walls. After data processing, two approaches were used for analysis of candidate genes, assuming that expression of these genes would have a similar trend to that of FA accumulation in seeds. First, we selected genes with at least a tenfold higher abundance in seeds of stage H5 than roots, leaves, and stems. In total, 816 genes were identified as seed-preferred genes, of which 69 were TFs (Fig. 1C, and Supplementary Table S1 at *JXB* online). Secondly, 750 genes with more than 1.5-fold higher abundance at stage H4 or H5 than other seed developmental stages were selected as H4/H5-preferred genes, including 18 new TF genes in addition to the seed-preferred TFs (Fig. 1D and Supplementary Table S1). We transformed these selected TF genes into *Arabidopsis* and examined the lipid content change in seeds of transgenic plants compared with that in wild-type plants. We focused on a bZIP TF gene, *GmbZIP123*, and further dissected its function in lipid accumulation. qRT-PCR analysis revealed that expression of *GmbZIP123* was highest in seeds at stage H5 and subsequently decreased at stage H6 (Fig. S1A at *JXB* online), in accordance with the results of RNA-seq (Fig. 1D). In addition to seeds, *GmbZIP123* was also expressed in all other examined organs and showed relatively high expression in roots (Fig. S1A).

### *GmbZIP123* sequence features

According to our previous classification in soybean bZIP TFs (Liao *et al*., 2008) and *Arabidopsis* bZIP TF analysis (Jakoby *et al*., 2002), GmbZIP123 belongs to group S bZIP TFs. Phylogenetic analysis of GmbZIP123 and *Arabidopsis* group S bZIP TFs revealed that GmbZIP123 clustered with group S1 bZIP TFs, including AtbZIP11, AtbZIP2, and AtbZIP44 (Fig. S1B). As group S1 bZIP TFs harbour the conserved upstream open reading frames (uORFs) in their 5′-untranslated region (UTR), which is translationally controlled by sucrose (Rook *et al*., 1998; Wiese *et al*., 2004), we examined whether the 5′UTR of *GmbZIP123* also contained uORFs. We used 5′RACE to clone the full-length gene sequence of *GmbZIP123* and found that two conserved uORFs existed in the 5′UTR of the *GmbZIP123* gene (Fig. S1C). The regulatory roles of these uORFs on *GmbZIP123* expression remain to be studied.

The protein sequence of GmbZIP123 was compared with those of *Arabidopsis* group S1 bZIP TFs. The bZIP domain was highly conserved in GmbZIP123 and *Arabidopsis* bZIP TFs, but the amino acid sequences of other regions shown much variation (Fig. S1D). The sequence divergence between GmbZIP123 and *Arabidopsis* group S1 bZIP TFs may suggest that *GmbZIP123* participates in different biological processes.

### Subcellular localization of GmbZIP123 and heterodimerization between GmbZIP123 and *Arabidopsis* group C bZIP TFs

**Fig. 2.**
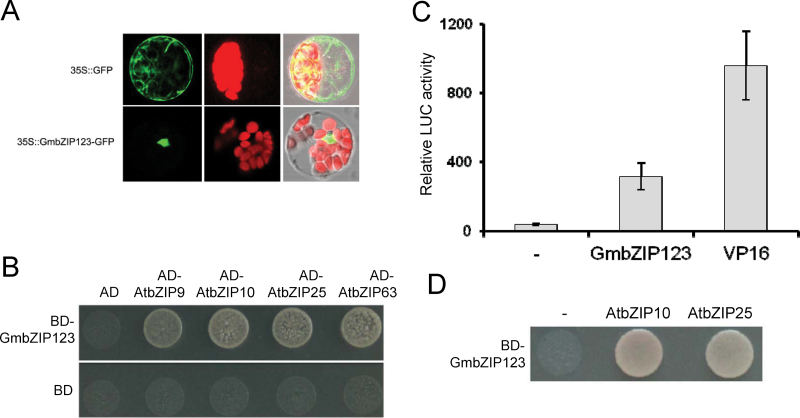
Subcellular localization, transcriptional activation, and interacting TFs of GmbZIP123. (A) Subcellular localization of GmbZIP123 in *Arabidopsis* protoplasts. (B) Protein interactions in the yeast two-hybrid system. BD empty vector was used as negative control. Growth of the transfected yeast cells on His-depleted medium indicates positive protein–protein interactions. (C) Transcriptional regulation activity of GmbZIP123 in a protoplast assay; – indicates empty vector and VP16 was used as a positive control. (D) GmbZIP123 interacts with AtbZIP10 and AtbZIP25 to induce reporter gene expression in yeast growing on His-depleted medium; – indicates empty vector.

To determine the subcellular location of GmbZIP123, *GmbZIP123* was fused to *GFP*, and the fusion gene under control of the 35S promoter was transformed into *Arabidopsis* protoplasts. GFP fluorescence was observed under a confocal microscope. GmbZIP123 was targeted exclusively to the nuclei of protoplast cells, while the control GFP protein was observed throughout the entire cell, demonstrating that GmbZIP123 is a nuclear protein (Fig. 2A).

Previous studies have demonstrated that group S bZIP TFs exert their functions through heterodimerization with group C bZIP TFs in higher plants (Ehlert *et al*., 2006). We examined whether GmbZIP123 could interact with *Arabidopsis* group C bZIP TFs by a yeast two-hybrid assay. There are four members of the *Arabidopsis* group C bZIP TFs, namely AtbZIP9, AtbZIP10, AtbZIP25, and AtbZIP63. *GmbZIP123* and the four *Arabidopsis* group C bZIP TFs were fused to the GAL4 BD or AD and expressed in yeast containing a *HIS3* reporter gene under the control of the GAL4 binding sites, respectively. Heterodimerization between GmbZIP123 and all *Arabidopsis* group C bZIP TFs was detected by prototrophic growth of the yeast strains on His-depleted medium, suggesting that GmbZIP123 could play roles in *Arabidopsis* through interaction with *Arabidopsis* group C bZIP TFs (Fig. 2B).

### Transcriptional regulation activity of GmbZIP123

*Arabidopsis* group S bZIP TFs possess transcription activation activity to promote expression of target genes (Hanson *et al*., 2008; Alonso *et al*., 2009; Ma *et al*., 2011). We further examined whether GmbZIP123 possessed transcription activation activity in transiently transformed *Arabidopsis* protoplasts. As shown in Fig. 2C, GmbZIP123 showed transcription activation activity to promote expression of a *LUC* reporter gene in protoplasts when compared with the negative control. However, this result seemed to be inconsistent with that obtained in the yeast assay (Fig. 2B), where a single BD–GmbZIP123 fusion protein could not activate transcription of the *HIS3* reporter gene (Fig. 2B). As many endogenous proteins are expressed in *Arabidopsis* protoplasts, the discrepancy of transcription activation activity in the yeast system and protoplast system was probably derived from the expression of GmbZIP123-interacting proteins in protoplasts. In view of the interaction between GmbZIP123 and *Arabidopsis* group C bZIP TFs, we proposed that GmbZIP123 may perform transcription activation mainly through heterodimerization with group C bZIP TFs. Therefore, we co-expressed GmbZIP123–BD fusion protein and AtbZIP10/AtbZIP25 in yeast, and the yeast strains could grow on SD/–His/–Trp medium, indicating that GmbZIP123 induces transcription of target genes through heterodimerization with group C bZIP TFs (Fig. 2D).

### Overexpression of *GmbZIP123* increases seed oil content in *Arabidopsis*

**Fig. 3.**
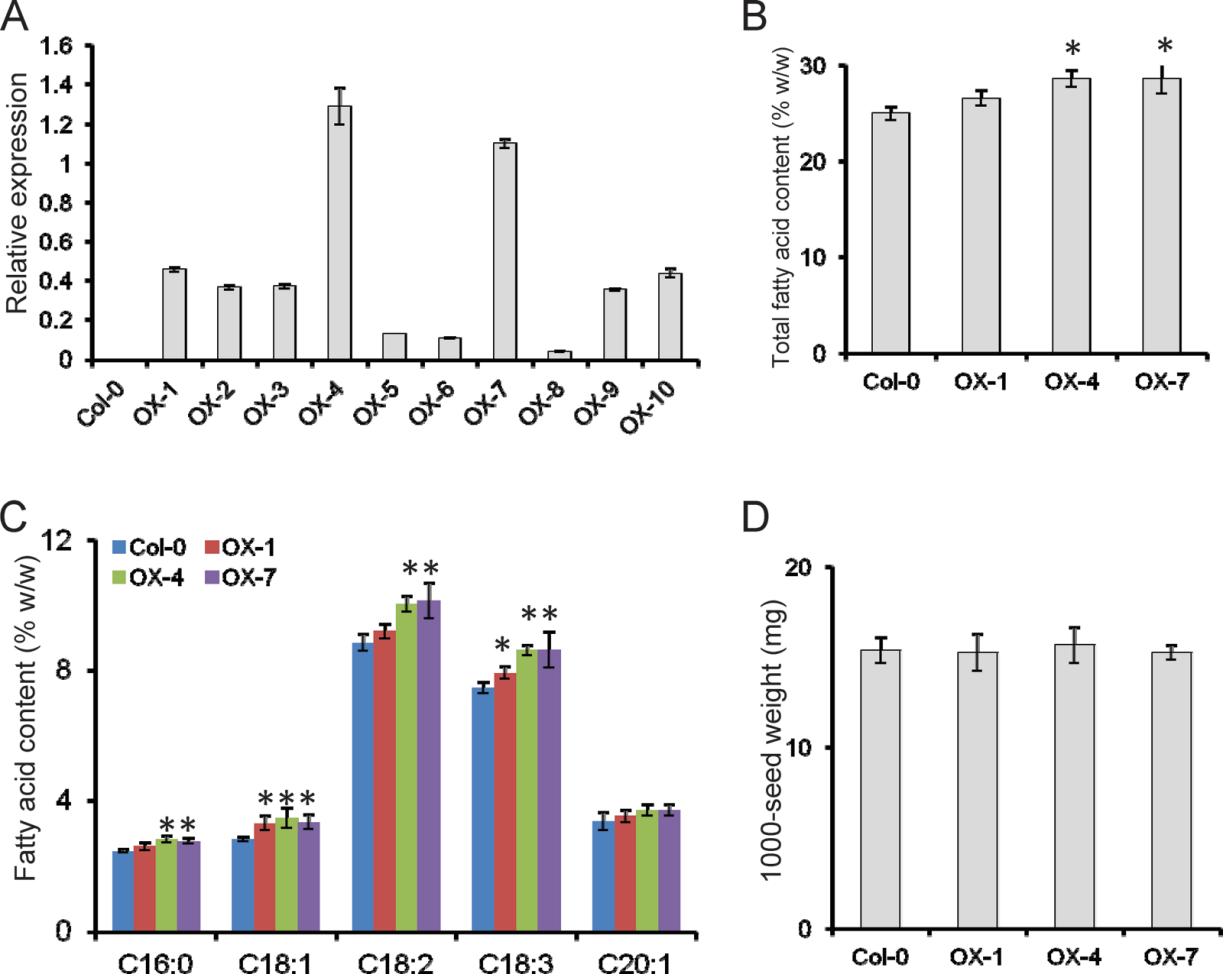
FA content in seeds of *GmbZIP123* transgenic *Arabidopsis* plants. (A) Expression of *GmbZIP123* in transgenic lines analysed by qRT-PCR using total RNA isolated from 2-week-old seedlings of Col-0 and *GmbZIP123* transgenic plants. The relative expression level of *GmbZIP123* was normalized using *AtACTIN2* as an internal control. For each column, bars indicate SD (*n*=3). (B) Total FA content in seeds of Col-0 and *GmbZIP123* transgenic plants analysed by GC analysis. (C) Major FA content in seeds of Col-0 and *GmbZIP123* transgenic plants. (D) The 1000-seed weights of Col-0 and *GmbZIP123* transgenic plants. For (B–D), results are shown as means±SD (*n*=4). Asterisk indicates significant differences compared with the control (one-way analysis of variance: *P* <0.05).

To investigate further the function of *GmbZIP123* in plants, we transformed *GmbZIP123* gene under the control of the 35S promoter into *Arabidopsis* and obtained ten transgenic *Arabidopsis* lines. After determination of *GmbZIP123* expression in transgenic lines by qRT-PCR, we selected three independent homozygous lines (OX-1, OX-4, and OX-7) with relatively high expression of *GmbZIP123* for further analysis (Fig. 3A). These transgenic plants did not show significant phenotypic changes compared with Col-0 plants (Fig. S2 at *JXB* online).

The FA content of mature seeds in *GmbZIP123* transgenic *Arabidopsis* lines was measured using GC. In comparison with wild-type plants, the total FA content of seeds increased by ~9–12% in overexpressing lines (OX-1, OX-4, and OX-7) (Fig. 3B). To examine whether the increase in total FA content in seeds of overexpressing lines was derived from the increase of one or several specific FA compositions, we analysed the content changes of major FAs (C16:0, C18:1, C18:2, C18:3, and C20:1) in the seeds of transgenic lines. We found that all examined FA species accumulated at a higher level in seeds of overexpressing lines (OX-1, OX-4, and OX-7) than in wild-type plants (Fig. 3C). The 1000-seed weight of transgenic lines was also measured and compared with wild-type plants. There was no significant change in 1000-seed weight between transgenic lines and wild-type plants (Fig. 3D). These results demonstrated that overexpression of *GmbZIP123* increases the FA content in seeds of transgenic plants and that this increase was not due to the accumulation of a specific FA composition, implying that *GmbZIP123* may function at the early steps of FA biosynthesis.

### Sugar metabolism genes are regulated by GmbZIP123

In order to explore the molecular mechanism of *GmbZIP123* in the control of FA synthesis, we attempted to identify genes that were differentially expressed between overexpressing lines and wild-type plants by RNA-seq. RNA prepared from seedlings of wild-type plants and two overexpressing lines (OX-1 and OX-4) at 16 d after germination were used for RNA-seq experiments. We defined genes as differentially expressed if their expression (reads per kb per million reads) had at least a 1.5-fold change in transgenic lines compared with wild-type plants and with a *P* value below 1e^–3^ (Fisher’s exact test). In total, 1980 genes were co-regulated in both the OX-1 and OX-4 lines, among which 800 genes were induced and 1180 genes were repressed (Supplementary Table S2 at *JXB* online).

As all examined FA species showed an increase in seeds of the *GmbZIP123* overexpressing lines, we first examined whether genes involved in *de novo* synthesis of FAs were induced by *GmbZIP123*. There are 51 annotated genes participating in *de novo* FA synthesis in plastids in *Arabidopsis* (Beisson *et al*., 2003). However, the expression of these 51 genes was not significantly upregulated in the overexpressing lines based on RNA-seq data, indicating that the FA content increase by *GmbZIP123* was not caused through elevated expression of enzyme genes that directly participated in FA synthesis. Previous studies have revealed that group S1 bZIP TFs exert broad functions in the metabolism of amino acids and sugars, and regulate expression of the related genes (Hanson *et al*., 2008; Alonso *et al*., 2009; Ma *et al*., 2011). A few of these genes, such as *ASPARAGINE SYNTHETASE1* (*ASN1*), *PROLINE DEHYDROGENASE* (*ProDH*), and *TREHALOSE-6-PHOSPHATE PHOSPHATASE 6* (*TPP6*), were also expressed at higher levels in both *GmbZIP123* overexpressing lines OX-1 and OX-4 compared with wild-type plants (Supplementary Table S2).

**Fig. 4.**
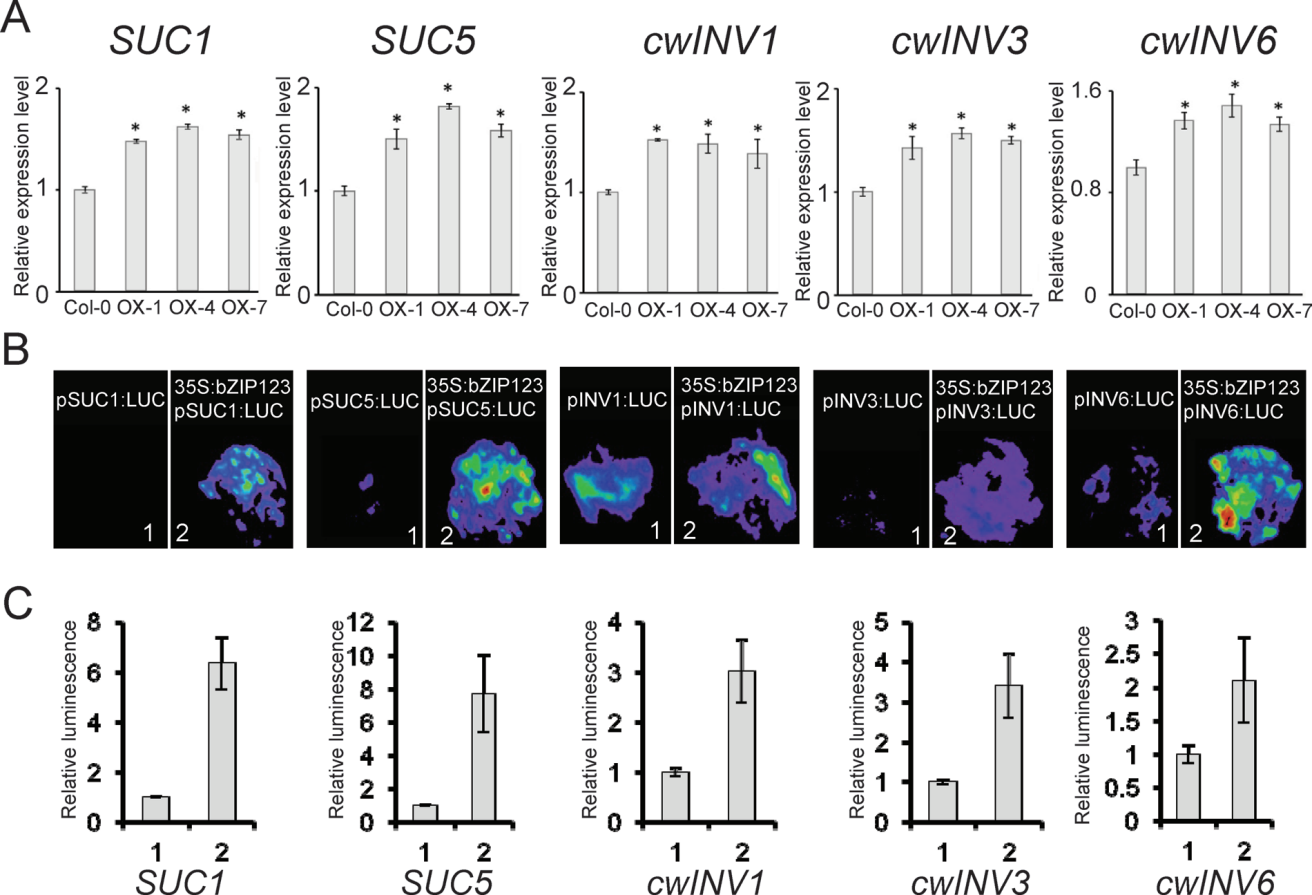
Altered gene expression regulated by *GmbZIP123*. (A) Relative expression levels of *GmbZIP123* regulated genes were analysed by qRT-PCR using total RNA isolated from 2-week-old seedlings of Col-0 and *GmbZIP123* transgenic plants. The relative expression level of each gene was normalized using *AtACTIN2* as an internal control. The transcription level of each gene in Col-0 was set as 1.0. For each column, bars indicate SD (*n*=3). Asterisks indicate significant differences compared with the control (one-way analysis of variance: *P* <0.05). (B) GmbZIP123 activated the expression of target genes by transient expression in tobacco leaves. (C) Quantitative analysis of luminescence intensity for each comparison in (B).

From the GmbZIP123-regulated genes (Supplementary Table S2), we found that five sucrose transport-related genes were significantly elevated by *GmbZIP123* overexpression, including *SUC1*, *SUC5*, *cwINV1*, *cwINV3*, and *cwINV6*. However, expression of other *SUC* and *cwINV* genes did not show significant alterations. Expression of these five sucrose transport-related genes was investigated further using qRT-PCR and their levels were significantly increased in the three *GmbZIP123* overexpressing lines compared with wild-type plants (Fig. 4A). These results indicated that GmbZIP123 may enhance FA content through upregulation of sugar metabolism-related genes.

Synthesis of FAs in seeds is tightly linked to sucrose transport into seeds from photoautotrophic tissues. The FA content increase in seeds of *GmbZIP123* overexpressing lines may be at least partially due to enhanced expression of sucrose transport-related genes. We then tested whether GmbZIP123 could activate expression of these five sucrose transport-related genes using a tobacco transient expression assay system. The 1200bp sequence upstream from the ATG codon of *SUC1*, *SUC5*, *cwINV1*, *cwINV3*, and *cwINV6* was fused to the *LUC* reporter gene and each of the constructs was co-transformed with the effector plasmid harbouring *35S*:*GmbZIP123* into tobacco leaves. In contrast to reporter vector control (Fig. 4B, panel 1 for each pair), inclusion of GmbZIP123 effector apparently activated expression of all the five genes as revealed from the increased LUC activities (Fig. 4B, C).

**Fig. 5.**
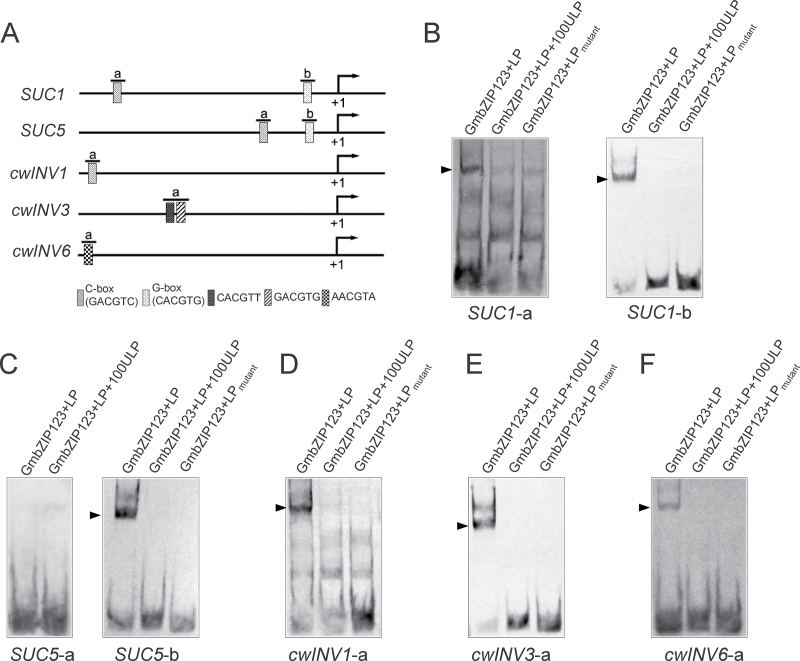
Binding of GmbZIP123 to the promoter of regulated genes. (A) Illustration of ACGT motif distribution in the promoters of *SUC1*, *SUC5*, *cwINV1*, *cwINV3*, and *cwINV6*. ‘a’ and ‘b’ were used to label different fragments containing an ACGT motif in the promoter region. The transcription start site is shown at position +1. (B–F) Gel-shift assay showing that GmbZIP123 can bind the promoter of *SUC1* (B), *SUC5* (C), *cwINV1* (D), *cwINV3* (E), and *cwINV6* (F). The probes in the gel-shift assay were derived from fragments labelled ‘a’ and ‘b’ in (A). The sequences of all probes are listed in Supplementary Table S4. LP, labelled probe; 100ULP, 100-fold unlabelled probe; LP_mutant_, labelled probe with mutated ACGT motif, which was changed to AAAA. Arrows indicate the position of the protein–DNA complex.

Plant bZIP TFs preferentially bind to DNA sequences containing ACGT elements, especially A-box (TACGTA), C-box (GACGTC), and G-box (CACGTG) sequences (Jakoby *et al*., 2002). The promoters of *SUC1*, *SUC5*, and *cwINV3* contain one or more C-box or G-box, while the promoters of *cwINV1* and *cwINV6* contain one or two other ACGT motifs (Fig. 5A). We next asked whether GmbZIP123 could directly bind to the promoters of these genes. The fragments covering ACGT motifs and flanking sequences in the 1200bp sequence upstream from the ATG codon were identified as candidate binding sites for each gene. GmbZIP123 was found to specifically bind to at least one fragment in upstream sequences of all these genes (Fig. 5B–F). However, GmbZIP123 could not bind to the mutated version of the fragments (Fig. 5B–F). These results indicated that GmbZIP123 probably regulates expression of *SUC1*, *SUC5*, *cwINV1*, *cwINV3*, and *cwINV6* by directly binding to the promoters of these genes.

### GmbZIP123 promotes cwINV activity and facilitates sugar translocation to siliques

**Fig. 6.**
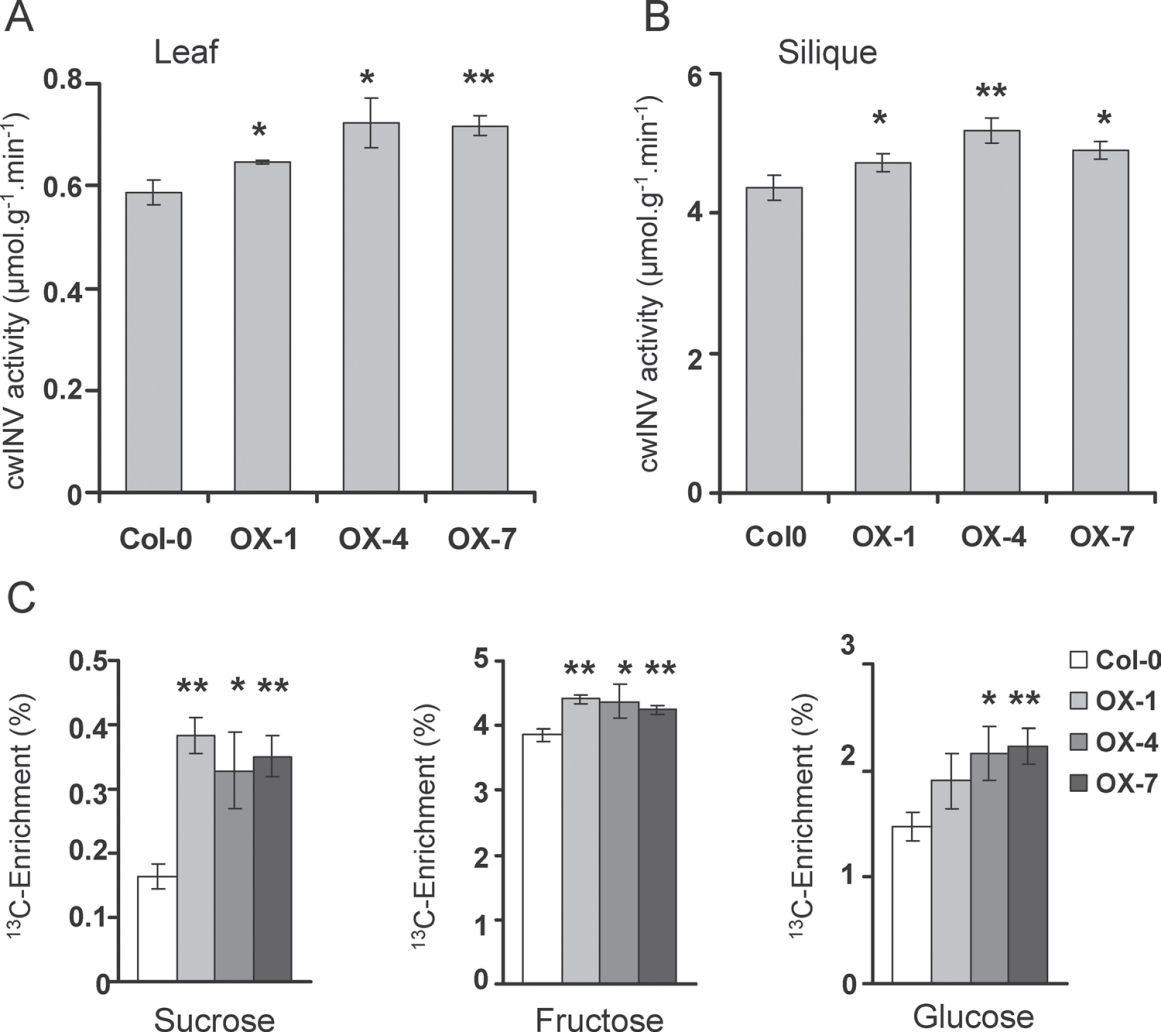
Comparison of cwINV activity and the level of sugar translocation in Col-0 and *GmbZIP123* transgenic lines. (A) cwINV activity in leaves of Col-0 and *GmbZIP123* transgenic lines. (B) cwINV activity in siliques of Col-0 and *GmbZIP123* transgenic lines. (C) ^13^C enrichment (dry weight) in sucrose, fructose, and glucose in siliques of Col-0 and *GmbZIP123* transgenic lines. For each column, bars indicate means±SD (*n*=4 for A and B; *n*=3 for C). Asterisks indicate a significant difference compared with the corresponding controls: *P <0.05 and **P <0.01.

SUCs are responsible for direct import of sucrose into cells, while cwINVs import sucrose by converting sucrose to hexoses and hexose transporters subsequently transport hexoses into cells. As the transcription of two SUCs (*SUC1* and *SUC5*) and three cwINVs (*cwINV1*, *cwINV3*, and *cwINV6*) were induced by *GmbZIP123*, we analysed cwINV activity and the level of sugar translocation from leaf to silique in Col-0 and *GmbZIP123* transgenic lines. Compared with Col-0, all three *GmbZIP123* transgenic lines displayed increases in cwINV activity in leaves and siliques (Fig. 6A, B). It should be also noted that cwINV activity was largely consistent with the *GmbZIP123* expression level (Fig. 3A).

To determine whether upregulation of *SUC* genes exerted a positive influence on sugar transport, 5-week-old plants were labelled with ^13^CO_2_ to monitor sugar translocation. The ^13^C enrichment levels of sucrose, fructose, and glucose in siliques of transgenic plants were all higher than that in Col-0 (Fig. 6C). These results indicated that increased allocation of labelled sugar flow into sink organ siliques was observed in the *GmbZIP123* transgenic lines.

### Sugar levels in seeds and silique walls of *GmbZIP123* transgenic plants

**Fig. 7.**
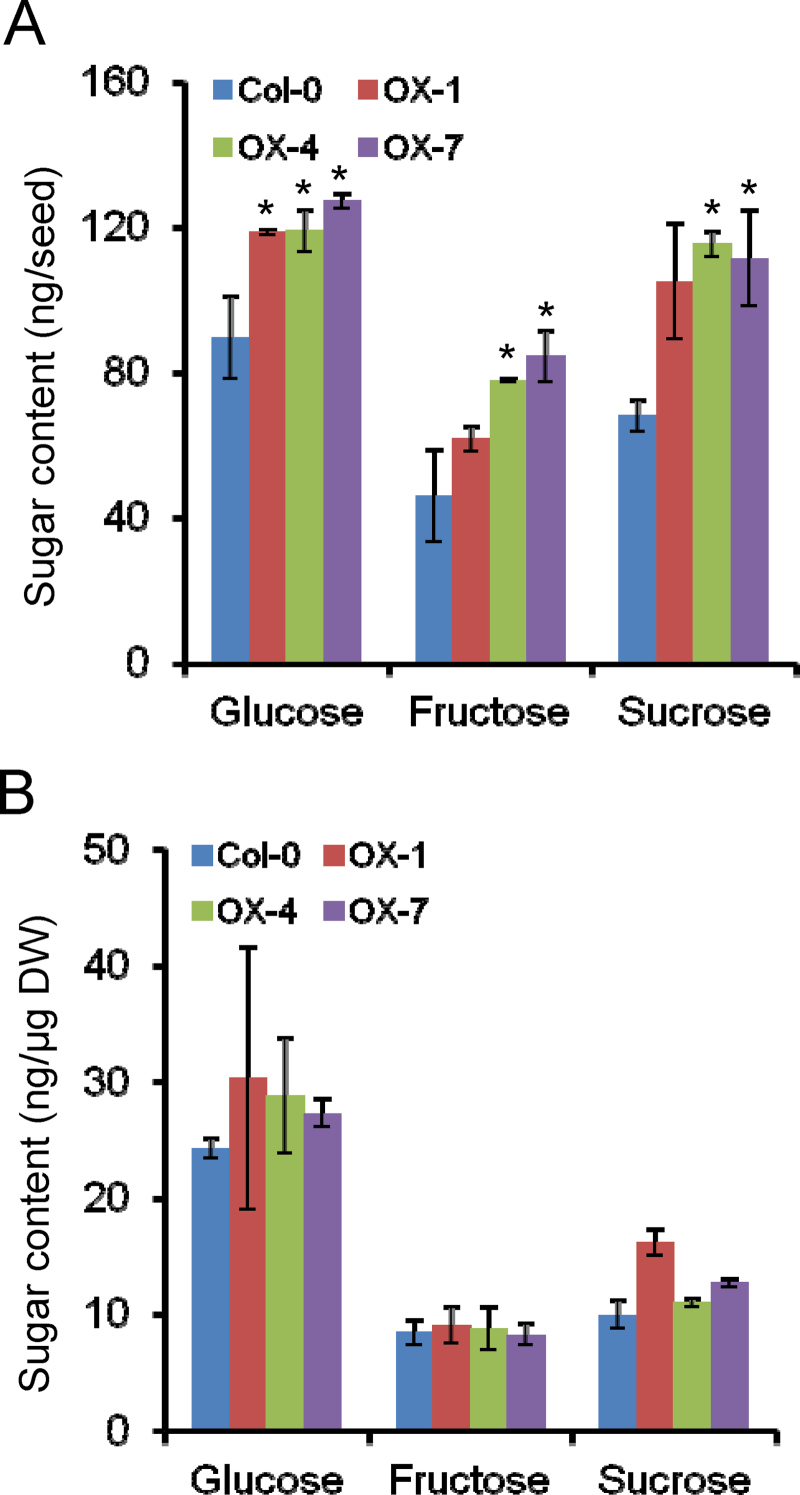
Comparison of glucose, fructose, and sucrose levels in developing seeds and silique walls of Col-0 and *GmbZIP123* transgenic lines. (A) Content of glucose, fructose, and sucrose in developing seeds of Col-0 and *GmbZIP123* transgenic lines. (B) Content of glucose, fructose, and sucrose in silique walls of Col-0 and *GmbZIP123* transgenic lines. The seeds and silique walls were both isolated from plants at 12 d after flowering. DW, dry weight. For each column, bars indicate means±SD (*n*=4). An asterisk indicates a significant difference compared with the corresponding controls at *P* <0.05.

Related to the analyses described above, we examined whether the *GmbZIP123* transgenic lines showed metabolic changes in sugars in developing silique walls and seeds at 12 d after flowering in transgenic plants by GC analysis. In developing seeds, glucose, fructose, and sucrose accumulated at higher levels in all transgenic lines than in wild-type plants (Fig. 7A). In silique walls, glucose and sucrose levels were mildly increased in transgenic plants, while the accumulation of fructose did not show an obvious change between transgenic and wild-type plants (Fig. 7B). These results suggested that more sugars are imported into seeds and silique walls of transgenic plants compared with wild-type plants, and GmbZIP123 may finally promote FA accumulation through activation of sugar translocation.

## Discussion

Lipid accumulation in plant seeds is a complex and well-organized biological process, accompanied by protein synthesis and starch consumption. A large amount of imported organic materials and energy are the indispensable foundation for rapid synthesis of lipid. Efforts to increase the lipid content of seeds should focus on how to enhance material import and accelerate the speed of lipid synthesis. In this study, we showed that overexpression of *GmbZIP123* in *Arabidopsis* could enhance lipid content in seeds of transgenic plants. Coincident with this observation, genes involved in sugar metabolism and transport were upregulated by *GmbZIP123*, and the cwINV activity and sugar transport were also enhanced in siliques of *GmbZIP123* transgenic plants. The levels of glucose, fructose, and sucrose were further increased significantly in seeds of transgenic plants. These results demonstrated that *GmbZIP123* participates in the regulation of lipid accumulation in seeds, probably through the promotion of sugar translocation.

As previous studies have shown that TFs exert broad functions in control of seed development and lipid synthesis (Focks and Benning, 1998; Tsukagoshi *et al*., 2007; Wang *et al*., 2007; Mu *et al*., 2008), we first made the effort to find candidate soybean TFs participating in regulation of lipid accumulation by identification of genes preferentially expressed at the stage of rapid lipid synthesis in soybean seeds. In total, 87 TFs were selected to further probe their functions in transgenic *Arabidopsis* plants (Supplementary Table S1). Further investigation of these TFs will facilitate an understanding of their functions in soybean seed development and lipid accumulation. In addition to *GmbZIP123*, we also studied the functions of other candidate TFs and some of these genes were found to play vital roles in seed development. It is worth noting that some seed-preferred TFs, consisting primarily of MADS-box TFs, showed high expression levels in silique wall (Fig. 1C, D, and Supplementary Table S1). In higher plants, MADS-box TFs can interact with other TFs to regulate many important biological processes, including the formation of flower organs, fruit development, and seed maturation (Vrebalov *et al*., 2002; Moyle *et al*., 2005; Ferrario *et al*., 2006). The growth of silique wall is ahead of seed development, and silique wall may provide large amounts of carbohydrate for rapid synthesis of lipid in seed. Research focusing on these MADS-box TFs may contribute to the exploration of molecular mechanisms involved in material exchange between the silique wall and seeds.

Phylogenetic analysis showed that GmbZIP123 belongs to group S1 bZIP TFs (Supplementary Fig. S1B). In *Arabidopsis*, group S1 bZIP TFs can interact with group C bZIP TFs to regulate the expression of target genes (Ehlert *et al*., 2006). GmbZIP123 itself did not show transcriptional activation activity in a yeast assay (Fig. 2B, D); however, GmbZIP123 could interact with *Arabidopsis* group C bZIP TFs to induce the expression of reporter gene in transfected cells, and these cells survived in selection medium in the yeast assay (Fig. 2D). These results indicated that GmbZIP123 requires other components for transcriptional activation, at least in yeast cells. However, it is unclear whether this phenomenon also happens in plant cells. The transcriptional activation ability in *Arabidopsis* protoplast assay (Fig. 2C) and in a transient expression assay in tobacco leaves (Fig. 4B, C), as well as activation of downstream genes related to sugar metabolism in *GmbZIP123* transgenic plants (Fig. 4A), suggested that GmbZIP123 can regulate target genes in plant systems. Further tests should be performed to investigate whether GmbZIP123 will need interaction with group C bZIP TFs for its functions in plant cells. Because of the complex interactions among bZIP TFs, GmbZIP123 may also interact with other bZIP TFs to exert various functions. Alternatively, another mechanism may also be involved in the *GmbZIP123*-regulated processes.

Due to the structural conservation among *Arabidopsis* group S1 bZIP TFs, some TFs could regulate common target genes. AtbZIP1, AtbZIP11, and AtbZIP53 bind directly to the promoters of *ASN1* and *ProDH* to induce their expression (Hanson *et al*., 2008; Alonso *et al*., 2009; Dietrich *et al*., 2011). Overexpression of *GmbZIP123* could also upregulate the expression of *ASN1* and *ProDH* in this study. Therefore, *GmbZIP123* may exert similar functions to *Arabidopsis* group S1 bZIP TFs in plants. However, different *Arabidopsis* group S1 bZIP TFs also participate in different biological processes. AtbZIP1 is a positive regulator of plant tolerance to salt, osmotic, and drought stresses (Kang *et al*., 2010; Sun *et al*., 2012). AtbZIP11 participates in trehalose metabolism by regulating expression of *TRE1*, *TPP5*, and *TPP6* (Hanson *et al*., 2008; Ma *et al*., 2011). Overexpression of *AtbZIP53* results in a dramatic increase in transcription of seed maturation genes in *Arabidopsis* leaves (Alonso *et al*., 2009). Because sequence divergence was found between GmbZIP123 and *Arabidopsis* group S1 bZIP TFs, GmbZIP123 probably regulates some specific target genes to play different roles in plants (Supplementary Fig. S1D). Consistent with this, GmbZIP123 actually regulates lipid accumulation in seeds through control of sugar transport and metabolism.

The increase in total FA levels in seeds of *GmbZIP123* transgenic plants was probably due to the elevation of each FA composition but not due to an increase in a specific FA composition (Fig. 3B, C), suggesting that the GmbZIP123 may act in the early steps of lipid biosynthesis. It has been reported that overexpression of a single gene in the ACCase or FAS complex could not significantly increase flux through FA biosynthesis; however, TFs have been identified as promoting expression of multiple genes in the ACCase or FAS complex to influence FA accumulation. For example, overexpression of *WRI1* upregulated a set of genes involved in FA synthesis in plastids, including *BCCP2*, *ACP1*, and *KAS1* (Baud *et al*., 2007). In the present study, GmbZIP123 may promote the flow and/or conversion of carbon to oil through modification of nutrient-loading cells or regulation of the transport machinery in the cell wall, including activation of genes (*SUC1*, *SUC5*, *cwINV1*, *cwINV3*, and *cwINV6*) involved in sugar transport but not activation of genes involved in plastidial FA synthesis. As an important carbon source of lipid synthesis, sugars should be efficiently supplied for rapid lipid synthesis in seeds. cwINVs catalyse sucrose into glucose and fructose, and play a significant role in sucrose unloading from the sieve elements (Wind *et al*., 2010). In *Arabidopsis* mutant of *SUC5*, FA concentration is reduced strongly in seeds at 8 d after flowering and is 2–13% lower in dry seeds than in wild-type seeds, suggesting that the expression of SUCs is very important for lipid synthesis in seeds (Baud *et al*., 2005). Recent research reports that SUC5 also functions in the delivery of biotin into the embryo to impact FA accumulation (Pommerrenig *et al*., 2013). Upregulated expression of *GmbZIP123* at the stage of rapid lipid synthesis in soybean seeds may enhance the import of sugars and/or biotin into seeds from photoautotrophic tissues, probably including leaves and silique walls (Fig. 1D), eventually facilitating lipid accumulation. This discovery provides a new insight into ways of increasing the lipid content of seeds. Higher levels of sugars in silique walls of transgenic plants may be due to more sugar import from leaves, because sucrose transporters (*SUC1* and *SUC5*) and cwINVs (*cwINV1*, *cwINV3*, and *cwINV6*) were also expressed in leaves. The elevated expression of these genes and the enzyme activity probably promoted sugar transport from leaves to silique walls and then to seeds. Rapid transport of sugars from source to sink may facilitate a feedback control for photosynthesis to produce more sugars in the source tissues. Although the regulatory mechanism of the source–sink network in plants is still elusive, these observations suggest that GmbZIP123 participates directly in the regulation of sugar transport in soybean.

It should be mentioned that, although the sugar content was increased in transgenic developing seeds (Fig. 7A), the 1000-seed weight was not significantly affected in *GmbZIP123* transgenic plants (Fig. 3D). The excess sugars may be prone to flux in lipid synthesis and the synthesis of other organic matter may then be influenced. Other possibilities may also be involved. Taken together, we found that overexpression of *GmbZIP123* enhanced FA content in transgenic seeds, probably through direct binding and activation of genes and promotion of sugar translocation. Our study provides novel insights into the regulatory mechanisms for lipid accumulation. Further comparison of the soybean and *Arabidopsis* systems in the transport pathway of the seed and anatomical and cell-wall differences should shed light on the regulatory roles of GmbZIP123 in lipid accumulation in soybean seeds.

## Supplementary data

Supplementary data are available at *JXB* online.

Supplementary Fig. S1. Expression pattern and sequence features of the soybean *GmbZIP123* gene.

Supplementary Fig. S2. Phenotype of Col-0 and *GmbZIP123* transgenic plants.

Supplementary Table S1. Candidate TFs participating in lipid accumulation.

Supplementary Table S2. List of differentially expressed genes in *GmbZIP123* transgenic plants.

Supplementary Table S3. Primers used in qRT-PCR.

Supplementary Table S4. Probes used in the gel-shift assay.

Supplementary Data

## Abbreviations:

5′RACE: rapid amplification of 5′ cDNA ends
ACCase: acetyl-CoA carboxylase
ACP: acyl carrier protein
AD: activation domain
BD: binding domain
bZIP: basic leucine zipper
cwINV: cell-wall invertase
FA: fatty acid
FAS: FA synthase
GC: gas chromatography
GFP: green fluorescent protein
LUC: luciferase
SD: standard deviation
SUC: sucrose transporter
TAG: triacylglycerols
TF: transcription factor
uORF: upstream open reading frames
UTR: untranslated region.
