# Supplementary material for: Soybean GmbZIP123 gene enhances lipid content in the seeds of transgenic Arabidopsis plants
Source: J Exp Bot. 2013 Aug 20;64(14):4329–41. doi: 10.1093/jxb/ert238 (PMC3808315; doi:10.1093/jxb/ert238)
Supplement: Supplementary Data [file supp_64_14_4329__index.html]

Soybean GmbZIP123 gene enhances lipid content in the seeds of transgenic Arabidopsis plants — Soybean GmbZIP123 gene enhances lipid content in the seeds of transgenic Arabidopsis plants — Supplementary Data 

# Soybean *GmbZIP123* gene enhances lipid content in the seeds of transgenic *Arabidopsis* plants

## 

Data files

**Files in this Data Supplement:**

- Supplementary Data - Supplementary Data
- Supplementary Data - Supplementary Data
- Supplementary Data - Supplementary Data
- Supplementary Data - Supplementary Data
- Supplementary Data - Supplementary Data
