# Supplementary material for: Soybean GmbZIP123 gene enhances lipid content in the seeds of transgenic Arabidopsis plants
Source: J Exp Bot. 2013 Aug 20;64(14):4329–41. doi: 10.1093/jxb/ert238 (PMC3808315; doi:10.1093/jxb/ert238)
Supplement: Supplementary Data [file supp_ert238_jexbot098277_file001.pdf]

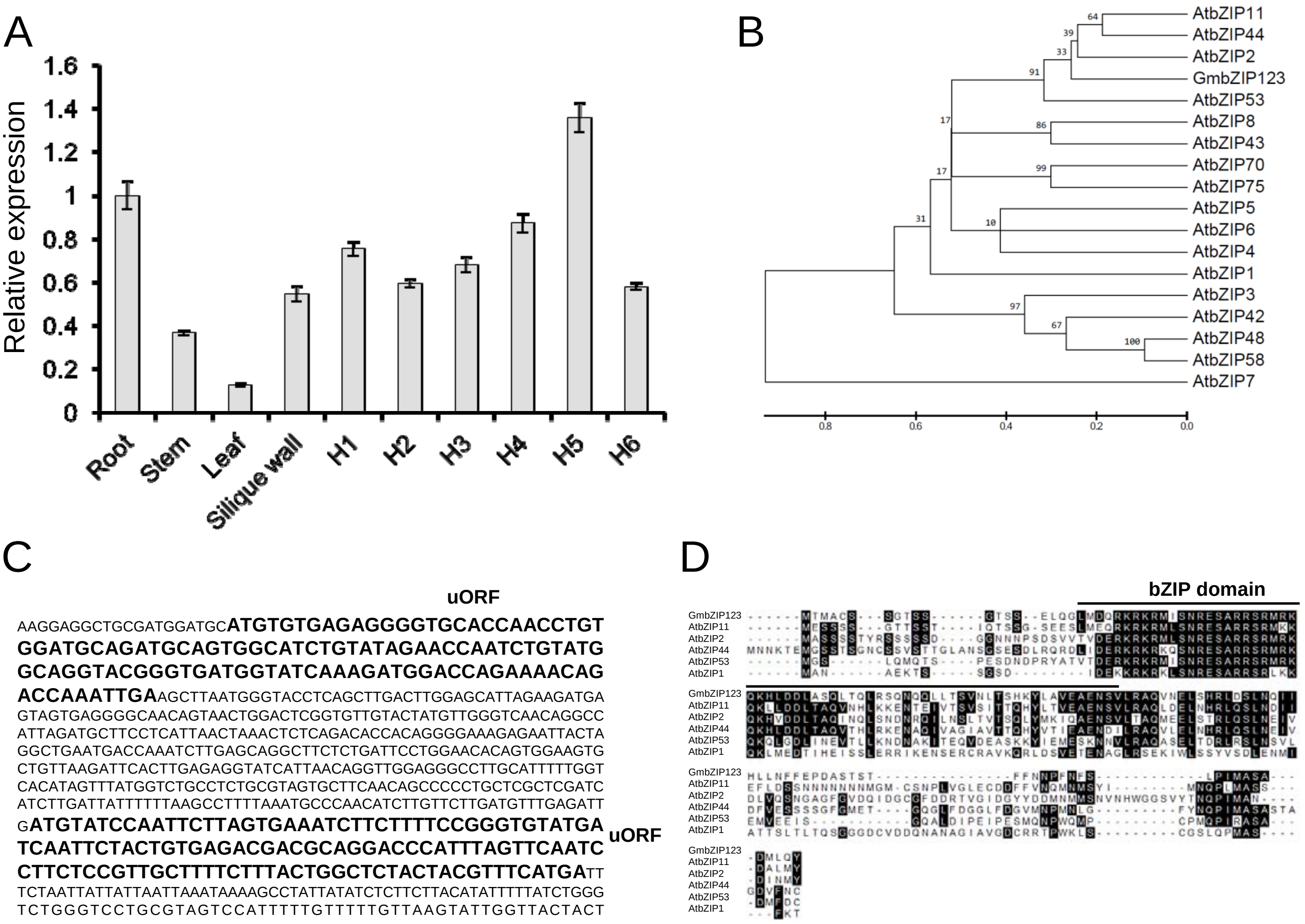

Figure S1. Expression pattern and sequence features of soybean *GmbZIP123* gene. A, Expression pattern of *GmbZIP123* in soybean various tissues analyzed by qRT-PCR. The relative expression level of *GmbZIP123* was normalized using *TUBULIN* as an internal control. B, Phylogenetic analysis of GmbZIP123 and Arabidopsis group S bZIP TFs. C, The 5'UTR sequence of *GmbZIP123* gene. uORF was highlighted in bold. D, Protein sequence alignment of GmbZIP123 and Arabidopsis group S1 bZIP transcription factors.

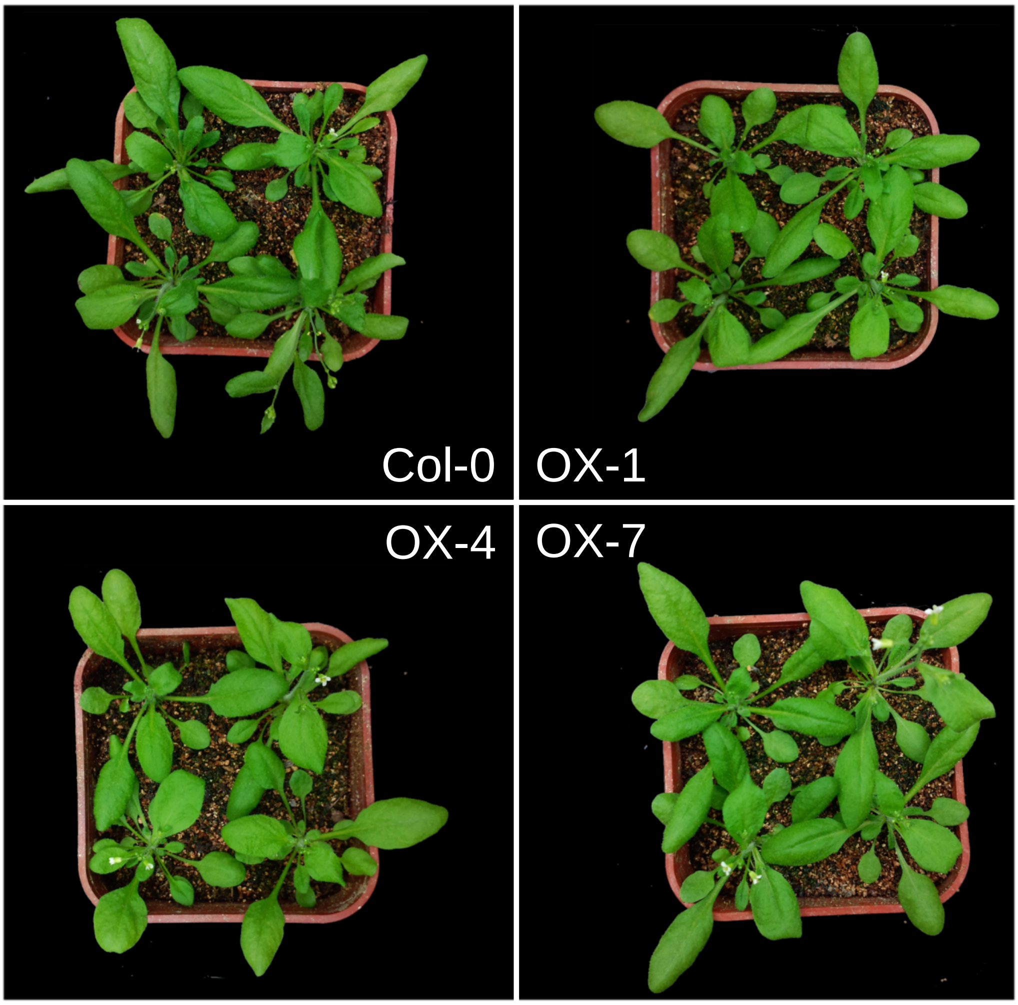

Figure S2. Phenotype of Col-0 and *GmbZIP123* transgenic plants (OX-1, 4, 7).
